# Discrepancy Between Clinician and Research Assistant in TIMI Score Calculation (TRIAGED CPU)

**DOI:** 10.5811/westjem.2014.9.21685

**Published:** 2014-11-11

**Authors:** Brian T. Taylor, Michelino Mancini

**Affiliations:** Lakeland HealthCare, Department of Emergency Medicine, St. Joseph MI, Department of Emergency Medicine, Saint Joseph, Michigan

## Abstract

**Introduction:**

Several studies have attempted to demonstrate that the Thrombolysis in Myocardial Infarction (TIMI) risk score has the ability to risk stratify emergency department (ED) patients with potential acute coronary syndromes (ACS). Most of the studies we reviewed relied on trained research investigators to determine TIMI risk scores rather than ED providers functioning in their normal work capacity. We assessed whether TIMI risk scores obtained by ED providers in the setting of a busy ED differed from those obtained by trained research investigators.

**Methods:**

This was an ED-based prospective observational cohort study comparing TIMI scores obtained by 49 ED providers admitting patients to an ED chest pain unit (CPU) to scores generated by a team of trained research investigators. We examined provider type, patient gender, and TIMI elements for their effects on TIMI risk score discrepancy.

**Results:**

Of the 501 adult patients enrolled in the study, 29.3% of TIMI risk scores determined by ED providers and trained research investigators were generated using identical TIMI risk score variables. In our low-risk population the majority of TIMI risk score differences were small; however, 12% of TIMI risk scores differed by two or more points.

**Conclusion:**

TIMI risk scores determined by ED providers in the setting of a busy ED frequently differ from scores generated by trained research investigators who complete them while not under the same pressure of an ED provider.

## INTRODUCTION

Chest pain is the second most common complaint of patients presenting to emergency departments (ED) in the United States, accounting for approximately seven million visits annually.^1^ Early determination of whether a patient’s chest pain origin is cardiac versus noncardiac is imperative. Patients diagnosed early with acute coronary diseases (ACS) may benefit from early interventions.^2^–^6^ A missed diagnosis of ACS may result in wrongful discharge, myocardial infarction and sudden death. Despite the use of electrocardiography (ECG) results, biomarker assays, patient history and clinical acumen, 0.4–5% of patients with acute myocardial infarction are inadvertently discharged from the ED.^7^–^14^

In an effort to improve outcomes in patients with acute coronary syndromes, researchers have developed numerous risk stratification tools.^15^–^57^ Of all the risk stratification systems developed, the thrombolysis in myocardial infarction (TIMI) risk score is the most studied, supported and used.^3^,^7^,^58^,^59^

A patient’s TIMI risk score is determined by assigning a value of one point for each of seven equally weighted prognostic variables with the total score determining a patient’s risk of adverse cardiac outcome (death, MI, severe recurrent ischemia requiring revascularization) within 14 days of presentation.

The TIMI risk score was originally derived from a retrospective analysis of a relatively high-risk population of patients with known unstable angina/non-ST elevation myocardial infarction.^15^ In this patient population the TIMI risk score was associated with 4.7% to 40.9% (or greater) risk of adverse cardiac outcome.^15^ Following the development of the TIMI risk score tool, several studies were performed validating the tool’s ability to stratify risk among patients with cardiac disease.^16^,^60^–^62^

Though not originally designed for ED use, several additional studies have attempted to demonstrate the TIMI risk score’s ability to stratify risk among real-world ED populations.^7^,^17^–^21^,^63^–^68^ As a result of these studies, the TIMI risk score tool has made its way into the protocols of EDs and hospitals around the world, often determining whether a patient is admitted to a hospital, observation unit or discharged home.^64^

### Importance

For many reasons, complete and accurate TIMI risk scores can be difficult to obtain when patients present with chest pain to a busy ED. Several studies have demonstrated how interruptions, distractions, and workload affect an ED provider’s ability to maintain thought flow and increase the likelihood of errors occurring.^69^–^72^ Pines et al.^73^ suggest that patients presenting to the ED during times of increased ED crowding are at greater risk for adverse cardiovascular outcomes. Inaccurate TIMI risk scores may result in inaccurate risk stratification, as well as ineffectual or inappropriate management of patients with nonspecific chest pain.

Most studies validating the utility of the TIMI risk score among ED populations used trained research investigators or a combination of trained researchers and ED providers to generate TIMI risk scores.^7^,^17^,^18^,^20^,^23^,^63^ Trained research investigators do not work under the same time constraints and in the same distracted environment as a working ED provider. Trained research investigators have the benefit of spending more time interviewing patients, reviewing medical records, scrutinizing ECG patterns, and reviewing their own scores for errors and clarification.^7^,^17^ Unfortunately, the ED provider does not usually have a trained research investigator at his or her disposal to determine accurate TIMI risk scores. Our review of the literature found very few prospective studies using ED providers exclusively as assessors for the TIMI risk score. In the select studies where ED providers assessed TIMI risk scores, their scores were not compared against those of trained study investigators for accuracy or validity.^64^,^65^

Current guidelines from the American College of Cardiology, American Heart Association, and National Institute for Health and Clinical Excellence strongly encourage the use of early risk stratification tools such as the TIMI risk score when patients present to healthcare providers with chest pain.^2^–^4^,^74^ In addition, Gallegher et al.^75^ suggest the possibility of medicolegal pitfalls by providers not using risk-stratifying tools when assessing patients for evidence of ACS. As a result, the TIMI risk score tool is increasingly being used by ED providers as a basis for therapeutic decision-making despite a lack of supporting studies using ED provider-obtained data.

### Outcomes of Interest

The primary goal of our study was to determine if TIMI risk scores obtained by ED providers in the setting of a busy ED differ substantially from those obtained by trained research investigators who complete them while not under the same pressure of a working ED provider. In addition, we evaluated whether ED provider type or patient gender had any effect on TIMI risk score discrepancy, which aspects of the TIMI risk score most frequently differ between assessors, and whether lower TIMI risk scores (i.e., 0–3) or higher TIMI risk scores (i.e., >3) more frequently match research investigator scores.

## METHODS

### Study Design

This was a prospective observational cohort study comparing TIMI scores obtained by ED providers admitting patients to the chest pain unit (CPU) at an academic-based community hospital to scores generated by trained research investigators. The local institutional review board approved the study without need for written informed consent.

### Study Setting and Population

Lakeland Regional Medical Center is an academic-based community hospital with an annual ED census of approximately 50,000 patients. The hospital’s six-bed CPU opened in 2010 and is situated adjacent to the ED. The CPU is open 24 hours a day, seven days a week and on holidays, with research investigators available 24 hours a day to enroll patients. The CPU is under the direct supervision of ED providers. All ED providers admitting patients to the CPU from October 27, 2012 until July 28, 2013 were included in the study. Participating ED providers included 18 attending physicians, 21 resident physicians and 10 midlevel providers (physician’s assistants and nurse practitioners). No ED providers were excluded from the study. Patient inclusion criteria included all comers presenting to the ED with non-traumatic chest pain suggestive of ACS who were admitted to our hospital’s CPU, irrespective of age. At our institution, ED providers independently determine who is to be placed in the CPU. Patient exclusion criteria for study enrollment mirrored CPU exclusion criteria as set by the hospital’s Chest Pain Center Door-to-Balloon Committee. Accordingly, patients with chest pain were excluded from admission to the CPU when any of the following were present:

- ST-elevation acute myocardial infarction (STEMI)
- Positive cardiac biomarkers suggestive of myocardial injury
- ECG changes
- Unrelenting chest pain
- Coronary revascularization in the last 60 days
- Abnormal vital signs
- New dysrhythmia (any run of ventricular dysrhythmia is not a candidate for the CPU)
- Aortic dissection
- Pneumothorax
- Pneumonia
- Esophageal rupture
- Pulmonary embolism
- Pericardial tamponade
- Congestive heart failure
- Uncontrolled diabetes
- Electrolyte abnormalities that could not be cared for with PO electrolyte replacement
- Psychiatric instability
- Inability to perform activities of daily living
- Pleural effusions
- Renal failure requiring dialysis during their time in the CPU
- Any diagnosis meeting admission criteria

### Study Protocol

**Figure 1:**
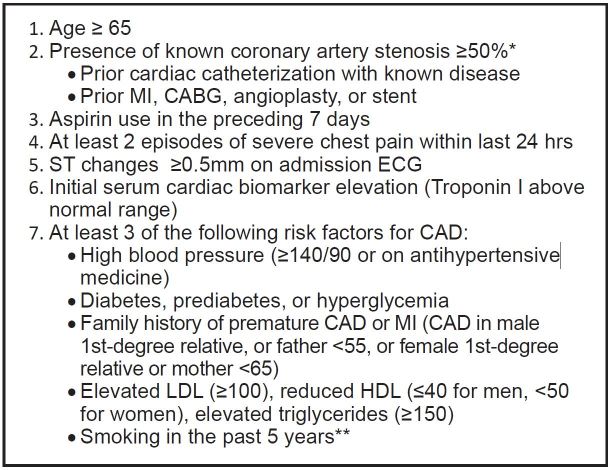
TIMI variables assessed by research investigators. *TIMI*, thrombolysis in myocardial infarction; *MI*, myocardial infarction; *CABG*, coronary artery bypass graft; *ECG*, electrocardiogram; *CAD*, coronary artery disease; *LDL*, low density lipoprotein; *HDL*, high density lipoprotein ^*^Similar to Pollack et al.,^65^ this parameter was expanded in our study because actual cardiac catheterization reports were not always available in the emergency department. ^**^5 years was chosen as a cut-off because risk associated with smoking has been found to diminish after 5 years.^76^–^78^

Research investigators consisted of registered CPU nurses who have completed formal ACS didactic sessions and learning modules. Prior to data collection, these research investigators received additional training on how to obtain TIMI risk scores. Their standardized training involved handouts, Microsoft Office PowerPoint presentations, and one-on-one training with clarification to increase the likelihood of unambiguous collection of data. Research investigators were instructed to use all resources available to them, including a patient’s hospital record, accessible outside records, labs, prior cardiac catheterization reports, cardiology notes, and patient-reported responses. Research investigators routinely evaluated the patient and assessed TIMI risk score variables within 24 hours of a patient’s presentation to the ED (Figure 1). In situations where patients were unaware or unable to answer questions concerning pertinent medical history (for example, an adopted patient unaware of his or her family history), patients were not given any points for those variables.

Our goal for the research investigator was not to obtain 100% infallible TIMI scores, but rather to generate scores as close as possible to scores assigned by research investigators performing similar TIMI risk-score validation studies.

Separately, ED providers assigned TIMI risk scores to all patients admitted to the CPU at the time of CPU admission per hospital protocol. No additional TIMI training or education was provided to ED providers prior to data collection. Research investigators and ED Providers were blinded to one other’s TIMI risk scores throughout the study.

### Data Analysis

Upon completion, we entered the pertinent data into an electronic database. We used SPSS software to make comparisons of TIMI risk scores obtained by research investigators and ED providers. Where significance testing was reported, we analyzed variables using the Pearson chi-square test.

## RESULTS

The patient population consisted of 543 patients who presented to the ED with symptoms suspicious for cardiac chest pain and were admitted to the CPU. Research investigators provided all variables used to form the TIMI risk score for 543 patients. ED providers provided the necessary variables for 501 patients. Because some ED providers did not record TIMI scores for every patient, we only had complete data for 501 patients. Of these 501 patients, 277 were female and 224 were male. The median age of the patient study population was 57 (ages 18 to 94).

Though the frequency distributions for research investigators and ED providers were similar, the two scores often did not match for a given patient (Table 1). In fact, of the 501 patients in the study with complete data, ED provider and researcher TIMI risk scores matched for only 213 patients (42.5%). Of the 213 patients with the same TIMI scores, only 147 scores (29.3%) were determined using identical TIMI variables. For example, one patient was given a TIMI score of one by both the research investigator and ED provider. On further analysis, however, the research investigator gave a point for aspirin use over the preceding seven days, while the ED provider gave a point for having three or more risk factors for CAD.

Further breakdown of TIMI scores revealed that scores differed by one point for 228 patients (45.5%), two points for 52 patients (10.4%), and three points for eight patients (1.6%). No scores varied by more than three points (Table 2).

Table 3 shows the incidence of TIMI variables as reported by research investigator and ED provider. The frequencies of several variables were similar, such as “Age ≥65”, “Aspirin use”, “ECG changes”, and “Elevated Troponin.” Research investigators reported a greater incidence of “Known CAD” and “Angina,” while ED providers reported a greater prevalence of “CAD Risk Factors.”

Our analysis showed that salient disagreements in TIMI variables existed between ED providers and research investigators. For example, ED providers reported the incidence “Angina” in only 59 of 207 patients (28.5%) determined by research investigators to have had “Angina”. Additionally, ED providers reported “Angina” as being present in 67 patients not reported by research investigators. Table 4 shows how often ED providers and research investigators agreed on reported variables.

**Figure 2:**
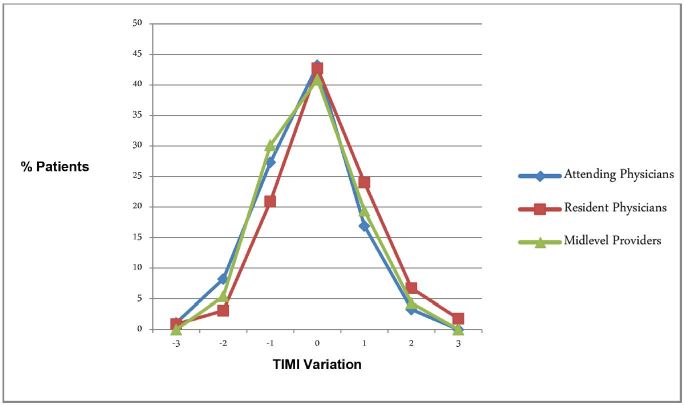
Range of TIMI score discrepancy from research investigator by ED provider type. *TIMI,* thrombolysis in myocardial infarction; *ED,* emergency department

We performed additional analysis based on ED provider type assessing the TIMI score (attending physician, resident physician or midlevel provider). Attending physicians determined the scores for 183 patients, resident physicians scored 225 patients, and midlevel providers scored 93 patients. Overall TIMI risk score determinations were similar across all provider types. TIMI scores matched 43.2% of researcher scores for attending physicians, 42.7% for resident physicians, and 40.9% for midlevel providers. When discrepancies occurred, attending physicians and midlevel providers reported slightly lower TIMI scores, while resident physicians reported slightly higher TIMI scores (Figure 2).

Further analysis showed that gender had little effect on TIMI score differences. ED provider scores agreed with research investigator scores for 112/277 female patients (40.4%) and for 103/224 male patients (46.0%).

Because the CPU at our institution is used to screen a population of patients at low-risk for ACS, far more low TIMI scores (TIMI 0–3) were generated. Based on the scores obtained by research investigators, 407 patients presenting to the CPU had TIMI scores 0–3, while only 94 had TIMI scores >3. There was no difference in the frequency of ED provider scores matching researcher scores on the basis of the number of variables involved (Table 5).

## DISCUSSION

This study demonstrated that a majority of TIMI scores as determined by ED providers in the setting of a busy ED differ from scores generated by trained research investigators who complete them while not under the same pressure of an ED provider. In our study only 29.3% of TIMI scores were calculated using identical TIMI risk score variables. The majority of TIMI risk score differences were either negligible (same TIMI risk score obtained despite differing TIMI variables used) or diverged by no more than one point in our low-risk patient population; however, 12% of patient scores differed by two or more points.

Our study examined a specific cohort of low-risk patients presenting to the ED with chest pain. CPU patients do not make up the entirety of patients presenting to the ED complaining of chest pain. Many times high-risk patients with ACS are admitted directly to the hospital or cath lab, and patients with noncardiac etiologies of chest pain (such as trauma or rash) are discharged home. Even though CPU populations make up a narrow range of the entire TIMI scale our data demonstrated a significant degree of variation between ED provider and trained research investigator scores. One might expect a greater degree of variation when using the whole spectrum of TIMI-derived risk scores.

We have shown that ED provider type has little effect on the likelihood of TIMI risk scores matching TIMI scores obtained by trained research investigators. Neither the patient gender nor the quantity of positive variables had a significant effect on TIMI risk score differences.

Patient age was the variable most agreed upon by TIMI risk score assessors with only one instance of an ED provider incorrectly giving a point to a 57-year-old for being ≥65 years old. TIMI variables requiring more active investigation showed greater variation. Researchers reported greater incidence of known CAD, possibly due to having more time available to review patient records and interview the patient. ED providers were apt to report a greater incidence of ≥3 CAD risk factors. Confirmation bias (or myside bias) is one potential reason for this. For example, in ascertaining the presence of multiple CAD risk factors (a time- consuming task), an ED provider might assume that when one or two risk factors are present, such as smoking and hypertension, other risk factors are likely present as well. Unfortunately, the TIMI risk score recorded in the electronic medical record by our ED providers simply shows when ≥3 CAD risk factors are present and does not further categorize which CAD risk factors were recognized by the ED provider.

Research investigators reported a few more instances of ECG and biomarker changes than were reported by ED providers. However, ECG changes and biomarker elevations were seldom present in our study, likely reflecting the low-risk nature of our CPU study population.

Both ED providers and research investigators reported similar numbers of aspirin users among our population; however, only 75.7% of these patients matched. Seventy-three patients recognized by ED providers as having taken aspirin went unrecognized by our research investigators. Likewise, research investigators reported an additional 58 patients whom ED providers said had not taken aspirin. Similar to aspirin, there was a discrepancy in the reporting of angina episodes. Researchers, who had the benefit of spending more time with patients, reported far more occurrences of angina than ED providers (207 to 126 occurrences). ED providers only recognized 59 of the 207 patients (28.5%) designated as having had angina by research investigators. Interestingly, ED providers reported angina as being present in 67 patients who research investigators did not feel met criteria for angina.

There are many barriers to obtaining accurate histories from patients.^79^–^81^ Patients who present to the ED in chest pain often do so under great duress, likely compounding the already difficult job of extracting accurate history. Studies have shown that patients in stressful situations have impairments in cognition, memory and verbal recall.^82^–^83^ Many clinicians recognize the phenomenon of the contradictory account, where the second person to interview a patient obtains an entirely different story. Perhaps in recognition of this, Hess et al.^17^ excluded patients with unreliable history from his prospective study on TIMI-score validity in the ED. The variability of patient-reported responses in the ED suggests a need for risk stratification tools which place greater weight on objective variables that can be assessed independently of interviews with the patient.

Many ED providers support the idea of using a clinical prediction rule for the identification of ACS among patients with chest discomfort in hopes of offering early discharge to low-risk patients.^84^ A few recent studies have suggested that a rapid TIMI risk score protocol can be employed to safely discharge low- risk ED patients with chest discomfort home from the ED.^22^,^23^,^84^ Though the TIMI risk score device has the potential to stratify risk among ED populations, our study suggests that it may depend on how and by whom the TIMI risk score data is obtained. In a study examining the use of a risk stratification tool commonly used in stroke management, Perry et al.^85^ demonstrated that ABCD2 scores calculated by ED physicians at bedside in the manner in which the score was intended to be used differed from scores calculated by trained research investigators, being lower for one-third of patients. It is important that any study suggesting validity and broad applicability of a risk-stratification tool for regular use in the ED, be examined closely to determine if the working data were obtained by ED providers while working in their normal environment. We commend validation studies such as Chase et al.^64^ and Pollack et al.^65^ for using ED providers to determine risk scores and call for more similar studies. We also question the applicability of studies that rely on data largely obtained by trained research investigators in place of ED providers.

Additional areas for future research may include investigating challenges particular to the application of risk-stratification tools in an ED environment, such as effects of ED crowding, ED provider staffing, and time restraints and distractions placed upon the ED provider. Studies examining the accuracy of patient-reported history in an ED environment may be useful in determining which elements of patient-recalled data can be reliably used in an ED-based risk-stratification tool. Furthermore additional studies comparing Attending level ED provider-obtained data to that of other ED attendings may be helpful in the evaluation of ED scoring accuracy.

## LIMITATIONS

Some researchers have suggested that ECG and biomarker indices should carry greater weight in risk- stratification scores.^17^,^40^ Modified TIMI risk scoring tools have been developed that assign more points to ECG and biomarker variables.^17^,^40^ Because so few ECG and biomarker changes were present in our study it is difficult to make generalizations on the ED provider’s ability to recognize and assign a proper TIMI risk score for those variables. Though not significant, the few ECG and biomarker changes recognized in our study were slightly underreported by ED providers, which may reflect a degree of selection bias or simply differences in interpretation. It is possible that ED providers under-report some aspects of the TIMI risk score (such as angina, ECG and biomarker changes) since they have already deemed a patient low risk and not likely suffering from true ACS by virtue of placing the patient in the CPU. In addition, ED providers may be less likely than research investigators to report a Troponin I level at the very edge of the cutoff as “positive,” especially in a patient with known chronic renal insufficiency, for example.

We asked our research investigators to obtain scores within 24 hours of patient presentation. This was done to improve the likelihood of obtaining complete data for the majority of patients. We recognize that research investigators in other studies may have had additional time to perform their investigations.

Research investigator TIMI risk score ECG interpretation was performed by our trained research investigators and not physicians well-versed in ECG interpretation. Additionally, CPU nurses have variable levels of clinical experience, which could have variable effects on TIMI scores, such as interpreting anginal chest pain. Had we included a second trained research investigator to determine a third TIMI score it is possible that differing scores may have resulted, thereby demonstrating further inter-assessor variability. Moreover, midlevel providers and resident physicians also have variable levels of training which could effect TIMI score variance.

Most data were acquired using information readily available to the research investigator in the CPU setting, which is similar to what is available to the ED provider. Data could sometimes be obtained via fax or telephone during regular business hours. Midway through the project some cardiologists released online access to their outpatient clinical electronic medical records, providing additional means of data acquisition.

Patient demographics may have also contributed to some study variation. Though predominantly English-speaking, our geographic area does contain some non-English speaking individuals, which could have impeded an assessor’s ability to obtain a reliable history.

## CONCLUSION

Our study demonstrates discordance between TIMI scores generated by trained research investigators and busy ED providers. Our study questions the reliability, validity, and applicability of previous TIMI risk score validation studies where scores were ascertained predominantly by trained research investigators.

## Figures and Tables

**Table 1:** Research investigator and ED provider TIMI scores.

TIMI score	Researcher (n)	ED provider (n)	ED provider score matches researcher score
0	96	99	54 (56.3%)
1	130	121	48 (36.9%)
2	92	109	34 (37.0%)
3	89	88	33 (37.1%)
4	71	70	38 (53.5%)
5	22	12	5 (22.7%)
6	1	2	1 (100%)
7	0	0	0 (100%)
Total patients	501	501	213 (42.5%)

*TIMI,* thrombolysis in myocardial infarction; *ED,* emergency department

**Table 2:** Discrepancy between emergency department provider and researcher TIMI scores.

Range of TIMI discrepancy	n	% of Total scores
−4	0	0
−3	4	0.8
−2	27	5.4
−1	125	25.0
0*	213	42.5
+1	103	20.6
+2	25	5.0
+3	4	0.8
+4	0	0
Total	501	100

*TIMI,* thrombolysis in myocardial infarction

* Matching

**Table 3:** Incidence of TIMI variables.

	Researcher n (%)	ED provider n (%)
Age ≥65	166 (33.1%)	167 (33.3%)
Known CAD	149 (29.7%)	118 (23.6%)
ASA use	239 (47.7%)	254 (50.7%)
Angina	207 (41.3%)	126 (25.1%)
ECG changes	9 (1.8%)	7 (1.4%)
Elevated trop	21 (4.2%)	10 (2.0%)
CAD risk factors	190 (37.9%)	274 (54.7%)

*TIMI,* thrombolysis in myocardial infarction; *ED*, emergency department; *CAD*, coronary artery disease; *ASA*, aspirin; *ECG*, electrocardiogram; *Trop*, troponin I cardiac biomarker

**Table 4:** TIMI variable agreement (ED provider variable matched research investigator variable for the same patient).

	Positive n (ED/R)	Negative n (ED/R)
Age ≥65	166/166 (100%)	334/335 (99.7%)
Known CAD	104/149 (69.8%)	338/352 (96.0%)
ASA use	181/239 (75.7%)	189/262 (72.1%)
Angina	59/207 (28.5%)	227/294 (77.2%)
ECG changes	2/9 (22.2%)	487/492 (99.0%)
Elevated trop	7/21 (33.3%)	477/480 (99.4%)
CAD risk factors	173/190 (91.1%)	210/311 (67.5%)

*TIMI,* thrombolysis in myocardial infarction ED, emergency department provider; R, research investigator; CAD, coronary artery disease; ASA, aspirin; ECG, electrocardiogram; Trop, troponin I cardiac biomarker

**Table 5:** TIMI risk score divergence by range.

TIMI risk score range	Researcher (n)	ED provider matches researcher TIMI score	Matching TIMI score with identical variables
0 to 3	407	169 (41.5%)	116 (28.5%)
4 to 6	94	44 (46.8%)	31 (33.0%)
Total	501	213 (42.5%)	147 (29.3%)

*TIMI,* thrombolysis in myocardial infarction; *ED, emergency department*

## References

[1] Niska R, Bhuiya F, Xu J (2010). National Hospital Ambulatory Medical Care Survey: 2007 Emergency Department Summary. National health Statistics reports.

[2] Jneid H, Anderson JL, Wright RS (2012). 2012 ACCF/AHA Focused Update of the Guideline for the Management of Patients With Unstable Angina/Non–ST-Elevation Myocardial Infarction (Updating the 2007 Guideline and Replacing the 2011 Focused Update): A Report of the American College of Cardiology Foundation/American Heart Association Task Force on Practice Guidelines. Circulation.

[3] Patel MR, Bailey SR, Bonow RO (2012). ACCF/SCAI/AATS/AHA/ASE/ASNC/HFSA/HRS/SCCM/SCCT/SCMR/STS 2012 Appropriate Use Criteria for Diagnostic Catheterization. Catheter Cardiovasc Interv.

[4] Hoekstra J, Cohen M (2009). Management of Patients with Unstable Angina/Non-ST-elevation Myocardial Infarction: A Critical Review of the 2007 ACC/AHA Guidelines. Int J Clin Pract.

[5] Mehta SR, Granger CB, Boden WE (2009). Early vs. delayed invasive intervention in acute coronary syndromes. N Engl J Med.

[6] Hirsch A, Windhausen F, Thijssen JGP (2007). Long-term outcome after an early invasive versus selective invasive treatment strategy in patients with non-STelevation acute coronary syndrome and elevated cardiac troponin T (the ICTUS trial): a follow-up study. Lancet.

[7] Holly J, Fuller M, Hamilton D (2013). Prospective evaluation of the use of the thrombolysis in myocardial infarction score as a risk stratification tool for chest pain patients admitted to an ED observation unit. Am J Emerg Med.

[8] Pope JH, Aufderheide TP, Ruthazer R (2000). Missed diagnoses of acute cardiac ischemia in the emergency department. N Engl J Med.

[9] Lindsell CJ, Anantharaman V, Diercks D (2006). EMCREG-International i^*^trACS Investigators. The Internet Tracking Registry of Acute Coronary Syndromes (i^*^trACS): a multicenter registry of patients with suspicion of acute coronary syndromes reported using the standardized reporting guidelines for emergency department chest pain studies. Ann Emerg Med.

[10] Dagnone E, Collier C, Pickett W (2000). Chest pain with nondiagnostic electrocardiogram in the emergency department: a randomized controlled trial of two cardiac marker regimens. CMAJ.

[11] Lee TH, Rouan GW, Weisberg MC (1987). Clinical characteristics and natural history of patients with acute myocardial infarction sent home from the emergency room. Am J Cardiol.

[12] McCarthy BD, Beshansky JR, D’Agostino RB (1993). Missed diagnoses of acute myocardial infarction in the emergency department: results from a multicenter study. Ann Emerg Med.

[13] Rouan GW, Hedges JR, Toltzis R (1987). A chest pain clinic to improve the follow-up of patients released from an urban university teaching hospital emergency department. Ann Emerg Med.

[14] Karounos M, Chang AM, Robey JL (2007). TIMI risk score: does it work equally well in both males and females?. Emerg Med J.

[15] Antman EM, Cohen M, Bernink PJ (2000). The TIMI risk score for unstable angina/non-ST elevation MI: A method for prognostication and therapeutic decision making. JAMA.

[16] Bartholomew BA, Sheps DS, Monroe S (2004). A population-based evaluation of the Thrombolysis in Myocardial Infarction Risk Score for unstable angina and non-ST elevation myocardial infarction. Clin Cardiol.

[17] Hess EP, Perry JJ, Calder LA (2010). Prospective validation of a modified thrombolysis in myocardial infarction risk score in emergency department patients with chest pain and possible acute coronary syndrome. Acad Emerg Med.

[18] Ramsay G, Podogrodzka M, McClure C (2007). Risk prediction in patients presenting with suspected cardiac pain: the GRACE and TIMI risk scores versus clinical evaluation. Q J Med.

[19] Tong KL, Kaul S, Wang XQ (2005). Myocardial contrast echocardiography versus Thrombolysis In Myocardial Infarction score in patients presenting to the emergency department with chest pain and a nondiagnostic electrocardiogram. J Am Coll Cardiol.

[20] Macdonald SP, Nagree Y, Fatovich DM (2011). Comparison of two clinical scoring systems for emergency department risk stratification of suspected acute coronary syndrome. Emerg Med Australas.

[21] Jaffery Z, Hudson MP, Jacobsen G (2007). Modified thrombolysis in myocardial infarction (TIMI) risk score to risk stratify patients in the emergency department with possible acute coronary syndrome. J Thromb Thrombolysis.

[22] Aldous SJ, Richards MA, Cullen L A New Improved Accelerated Diagnostic Protocol Safely Identifies Low-risk Patients With Chest Pain in the Emergency Department. Acad Emerg Med.

[23] Than M, Cullen L, Reid CM (2011). A 2-h diagnostic protocol to assess patients with chest pain symptoms in the Asia-Pacific region (ASPECT): a prospective observational validation study. Lancet.

[24] Six AJ, Backus BE, Kelder JC (2008). Chest pain in the emergency room: value of the HEART score. Neth Heart J.

[25] Limkakeng A, Gibler WB, Pollack C (2001). Combination of Goldman risk and initial cardiac troponin I for emergency department chest pain patient risk stratification. Acad Emerg Med.

[26] Baxt WG (1991). Use of an artificial neural network for the diagnosis of myocardial infarction. Ann Intern Med.

[27] Baxt WG, Shofer FS, Sites FD (2002). A neural computational aid to the diagnosis of acute myocardial infarction. Ann Emerg Med.

[28] Baxt WG, Skora J (1996). Prospective validation of artificial neural network trained to identify acute myocardial infarction. Lancet.

[29] Goldman L, Weinberg M, Weisberg M (1982). A computer-derived protocol to aid in the diagnosis of emergency room patients with acute chest pain. N Engl J Med.

[30] Goldman L, Cook EF, Brand DA (1988). A computer protocol to predict myocardial infarction in emergency department patients with chest pain. N Engl J Med.

[31] Goldman L, Cook EF, Johnson PA (1996). Prediction of the need for intensive care in patients who come to the emergency departments with acute chest pain. N Engl J Med.

[32] Pozen MW, D’Agostino RB, Selker HP (1984). A predictive instrument to improve coronary-care-unit admission practices in acute ischemic heart disease. A prospective multicenter clinical trial. N Engl J Med.

[33] Selker HP, Beshansky JR, Griffith JL (1998). Use of the acute cardiac ischemia time-insensitive predictive instrument (ACI-TIPI) to assist with triage of patients with chest pain or other symptoms suggestive of acute cardiac ischemia. A multicenter, controlled clinical trial. Ann Intern Med.

[34] Christenson J, Innes G, McKnight D (2006). A clinical prediction rule for early discharge of patients with chest pain. Ann Emerg Med.

[35] Campbell CF, Chang AM, Sease KL (2009). Combining Thrombolysis in Myocardial Infarction risk score and clear-cut alternative diagnosis for chest pain risk stratification. Am J Emerg Med.

[36] Rubinshtein R, Halon DA, Gaspar T (2007). Usefulness of 64-slice cardiac computed tomographic angiography for diagnosing acute coronary syndromes and predicting clinical outcome in emergency department patients with chest pain of uncertain origin. Circulation.

[37] Hollander JE, Chang AM, Shofer FS (2009). Coronary computed tomographic angiography for rapid discharge of low-risk patients with potential acute coronary syndromes. Ann Emerg Med.

[38] Hollander JE, Chang AM, Shofer FS (2009). One year outcomes following coronary computerized tomographic angiography for evaluation of emergency department patients with potential acute coronary syndrome. Acad Emerg Med.

[39] Hoffmann U, Bamberg F, Chae CU (2009). Coronary computed tomography angiography for early triage of patients with acute chest pain: the ROMICAT (Rule Out Myocardial Infarction using Computer Assisted Tomography) trial. J Am Coll Cardiol.

[40] Body R, Carley S, McDowell G (2009). Can a modified thrombolysis in myocardial infarction risk score outperform the original for risk stratifying emergency department patients with chest pain?. Emerg Med J.

[41] Gatien M, Perry JJ, Stiell IG (2007). A clinical decision rule to identify which chest pain patients can safely be removed from cardiac monitoring in the emergency department. Ann Emerg Med.

[42] Kim JH, Jeong MH, Ahn Y (2011). A novel risk stratification model for patients with non-ST elevation myocardial infarction in the Korea Acute Myocardial Infarction Registry (KAMIR): Limitation of the TIMI risk scoring system. Chonnam Med J.

[43] Carmo P, Ferreira J, Aguiar C (2011). Does continuous ST-segment monitoring add prognostic information to the TIMI, PURSUIT, and GRACE risk scores?. Ann Noninvasive Electrocardiol.

[44] Gonclaves PA, Ferreira J, Aguiar C (2005). TIMI, PURSUIT, and GRACE risk scores: sustained prognostic value and interaction with revascularization in NSTE-ACS. Eur Heart J.

[45] Bracco C, Melchio R, Sturlese U (2010). Early stratification of patients with chest pain and suspected acute coronary syndrome in the Emergency Department. Minerva Med.

[46] Zairis MN, Lyras AG, Makrygiannis SS (2005). Continuous 12-lead electrocardiographic ST monitoring adds prognostic information to the Thrombolysis In Myocardial Infarction Risk Score in patients with non-ST-elevation acute coronary syndromes. Clin Cardiol.

[47] Manenti ERF, Bodanese LC, Camey SA (2006). Prognostic value of serum biomarkers in association with TIMI risk score for acute coronary syndromes. Clin Cardiol.

[48] Eagle KA, Lim MJ, Dabbous OH, GRACE Investigators (2004). A validated prediction model for all forms of acute coronary syndrome: estimating the risk of 6-month postdischarge death in an international registry. JAMA.

[49] Boersma E, Pieper KS, Steyerberg EW, for the PURSUIT Investigators (2000). Predictors of outcome in patients with acute coronary syndromes without persistent ST-segment elevation. Results from an international trial of 9461 patients. Circulation.

[50] Kurz DJ, Bernstein A, Hunt K (2009). Simple point of care risk stratification in acute coronary syndromes: The AMIS model. Heart.

[51] Piombo AC, Gagliardi JA, Guetta J (2003). A new scoring system to stratify risk in unstable angina. BMC Cell Biol.

[52] Singh M, Reeder GS, Jacobsen SJ (2002). Scores for post-myocardial infarction risk stratification in the community. Circulation.

[53] Morrow DA, Antman EM, Giugliano RP (2001). A simple risk index for rapid initial triage of patients with ST-elevation myocardial infarction: an InTIME II substudy. Lancet.

[54] Soderholm M, Deligani MM, Choudhary M (2012). Ability of risk scores to predict a low complication risk in patients admitted for suspected acute coronary syndrome. Emerg Med J.

[55] Chandra A, Lindsell CJ, Limkakeng A (2009). Emergency Physician High Pretest Probability for Acute Coronary Syndrome Correlates with Adverse Cardiovascular Outcomes. Acad Emerg Med.

[56] Diercks DB, Hollander JE, Sites F (2004). Derivation and Validation of a Risk Stratification Model to Identify Coronary Artery Disease in Women Who Present to the Emergency Department with Potential Acute Coronary Syndromes. Acad Emerg Med.

[57] Hoekstra JW, Pollack CV, Roe MT (2002). Improving the Care of Patients with Non-ST-elevation Acute Coronary Syndromes in the Emergency Department: The CRUSADE Initiative. Acad Emerg Med.

[58] Vadeboncoeur A, Dankoff J, Lang ES (2009). Chest pain, in evidence-based Emergency Medicine.

[59] Dunham M, Challen K, Walter D (2010). Risk stratification of patients with acute chest pain without a rise in troponin: current practice in England. Emerg Med J.

[60] Morrow DA, Antman EM, Snapinn SM (2002). An integrated clinical approach to predicting the benefit of tirofiban in non-ST elevation acute coronary syndromes: application of the TIMI risk score for UA/NSTEMI in PRISM-PLUS. Eur Heart J.

[61] Cannon CP, Weintraub WS, Demopoulos LA (2001). Comparison of early invasive and conservative strategies in patients with unstable coronary syndromes treated with the glycoprotein IIb/IIIa inhibitor Tirofiban. N Engl J Me.

[62] Aragam KG, Tamhane UU, Kline-Rogers E (2009). Does simplicity compromise accuracy in ACS risk prediction? A retrospective analysis of the TIMI and GRACE risk scores. PLoS One.

[63] Weisenthal BM, Chang AM, Walsh KM (2010). Relation between Thrombolysis in Myocardial Infarction Risk Score and one-year outcomes for patients presenting at the Emergency Department with potential acute coronary syndrome. Am J Cardiol.

[64] Chase M, Robey JL, Zogby KE (2006). Prospective validation of the Thrombolysis in Myocardial Infarction Risk Score in the emergency department chest pain population. Ann Emerg Med.

[65] Pollack CV, Sites FD, Shofer FS (2006). Application of the TIMI risk score for unstable angina and non-ST elevation acute coronary syndrome to an unselected emergency department chest pain population. Acad Emerg Med.

[66] Hess EP, Agarwal D, Chandra S (2010). Diagnostic accuracy of the TIMI risk score in patients with chest pain in the emergency department: a meta-analysis. CMAJ.

[67] Lee B, Chang AM, Matsuura AC (2011). Comparison of cardiac risk scores in ED patients with potential acute coronary syndrome. Crit Pathw Cardiol.

[68] Lyon R, Morris AC, Caesar D (2007). Chest Pain presenting to the Emergency Department—to stratify risk with GRACE or TIMI?. Resuscitation.

[69] Chisholm CD, Collison EK, Nelson DR (2000). Emergency department workplace interruptions: are emergency physicians “interrupt-driven” and “multitasking”?. Acad Emerg Med.

[70] Chisholm CD, Dornfeld AM, Nelson DR (2001). Work Interrupted: a comparison of workplace interruptions in emergency departments and primary care offices. Ann Emerg Med.

[71] Laxmisan A, Hakimzada F, Sayan OR (2007). The Multitasking clinician: decision-making and cognitive demand during and after team handoffs in emergency care. Int J Med Inform.

[72] Rivera AJ, Karsh BT (2010). Interruptions and Distractions in Healthcare: Review and Reappraisal. Qual Sal Heath Care.

[73] Pines JM, Pollack CV (2009). The association between Emergency Department crowding and adverse cardiovascular outcomes in patients with chest pain. Acad Emerg Med.

[74] Camm J, Gray H, Antoniou S (2010). Unstable angina and NSTEMI: the early management of unstable angina and non-ST-segment-elevation myocardial infarction. NICE Clinical Guideline.

[75] Gallagher S, Knight C, Wragg A (2010). Medicolegal pitfalls in the management of chest pain. Clin Risk.

[76] Critchley JA, Capewell S (2003). Smoking cessation for the secondary prevention of coronary heart disease. Cochrane Database Syst Rev.

[77] Anthonisen NR, Skeans MA, Wise RA (2005). The effects of a smoking cessation intervention on 14.5-year mortality: a randomized clinical trial. Ann Intern Med.

[78] Doll R, Peto R, Boreham J (2004). Mortality in relation to smoking: 50 years’ observations on male British doctors. BMJ.

[79] Yoon PW, Scheuner MT, Peterson-Oehlke KL (2002). Can family history be used as a tool for public health and preventive medicine?. Genet Med.

[80] Daelemans S, Vandevoorde J, Vansintejan J (2013). The Use of Family History in Primary Health Care: A Qualitative Study. Adv Prev Med.

[81] Fuller M, Myers M, Webb T (2010). Primary care providers’ responses to patient-generated family history. J Genet Couns.

[82] Olver JS, Pinney M, Maruff P Impairments of Spatial Working Memory and Attention Following Acute Psychosocial Stress. Stress Health.

[83] Hidalgo V, Almela M, Villada C (2014). Acute stress impairs recall after interference in older people, but not in young people. Horm Behav.

[84] MacGougan CK, Christenson JM, Innes GD (2001). Emergency physicians’ attitudes toward a clinical prediction rule for the identification and early discharge of low risk patients with chest discomfort. CJEM.

[85] Perry JJ, Sharma M, Sivilotti ML (2011). Prospective validation of the ABCD2 score for patients in the emergency department with transient ischemic attack. CMAJ.

